# Becoming Endemic: Anaplasmosis Imported Across State Borders

**DOI:** 10.7759/cureus.57902

**Published:** 2024-04-09

**Authors:** Joseph D Abraham, Heather S Wenning, Daniyal A Saeed, Armo Derbarsegian, Barry A Brook, Peimei He

**Affiliations:** 1 Internal Medicine, The Jewish Hospital – Mercy Health, Cincinnati, USA; 2 Medicine, University of Cincinnati College of Medicine, Cincinnati, USA; 3 Infectious Diseases, The Jewish Hospital – Mercy Health, Cincinnati, USA

**Keywords:** co-infection, epidemiology, leukopenia, thrombocytopenia, tick-borne, anaplasmosis

## Abstract

*Anaplasma phagocytophilum* is the causative agent of human granulocytic anaplasmosis (HGA), a tick-borne illness with increasing incidence since being described in the 1990s. Importantly, the presentation can be vague, yet prompt treatment is paramount. An 81-year-old Caucasian female was hospitalized in Cincinnati, Ohio, for fever and confusion following prolonged outdoor exposure in Emlenton, Pennsylvania. She initially was treated for sepsis from presumed community-acquired pneumonia; however, the combination of leukopenia, thrombocytopenia, and elevated liver enzymes prompted empiric tick-borne illness consideration and treatment with rapid resolution in symptoms. Early recognition of HGA can reduce unnecessary treatments and improve patient outcomes.

## Introduction

Human granulocytic anaplasmosis (HGA) is caused by the Gram-negative intracellular bacteria *Anaplasma phagocytophilum* [1]. HGA, formerly known as human granulocytic ehrlichiosis, is transmitted by the *Ixodes scapularis *tick and is commonly reported in the Midwest and the Northeast United States, with most cases occurring in June and July [2,3].

Anaplasmosis typically presents with non-specific symptoms, including fever, malaise, myalgia, arthralgia, headache, and general gastrointestinal symptoms, after one to two weeks [4]. Rarely, it may present with rash or nervous system involvement. Laboratory abnormalities include leukopenia, thrombocytopenia, elevated liver enzymes, and elevated serum creatinine. Peripheral blood smear may reveal morulae, which are intracytoplasmic aggregates within neutrophils [5]. Severe illness is rare but may cause renal or respiratory failure, peripheral neuropathy, coagulopathy, rhabdomyolysis, hemorrhage, or hemophagocytic lymphohistiocytosis [6]. First-line treatment is doxycycline [7].

This is an imported case of HGA that was initially treated as community-acquired pneumonia. Careful consideration of patient history and laboratory analysis led to tick-borne illness consideration and diagnosis in an HGA non-prevalent region.

## Case presentation

An 81-year-old Caucasian female presented to the hospital with new-onset confusion. Prior to her arrival, she spent a week in Emlenton, Pennsylvania, in a cabin with her husband. Symptoms started four days before admission consisting of fever, fatigue, headache, myalgias, diffuse joint pain, emesis, and non-bloody diarrhea. She reported a fever of 104.1°F one day prior to presentation. There were no known animal contacts, tick bites, or sick contacts.

On presentation, the patient was afebrile, hemodynamically stable, somnolent, and only oriented to self. Her physical examination was notable for diminished bilateral breath sounds with left-sided rales and abdominal distention; no dermatologic abnormalities were identified.

Her laboratory workup in the emergency department (ED) revealed leukocytopenia, thrombocytopenia, elevated liver enzymes, and acute kidney injury (AKI) (Table 1).

**Table 1:** Laboratory Values in the ED

Parameter	Result	Reference Range
White blood cell count	3.3 K/uL	4.0 - 11.0 K/uL
Platelet count	29 K/uL	135 - 450 K/uL
Alanine transaminase (ALT)	126 U/L	10 - 40 U/L
Aspartate aminotransferase (AST)	214 U/L	15 - 37 U/L
Lipase	138.0 U/L	13.0 - 60.0 U/L
Serum sodium	130 mmol/L	136 - 145 mmol/L
Serum potassium	2.6 mmol/L	3.5 - 5.1 mmol/L
Serum chloride	85 mmol/L	99 - 110 mmol/L
Blood urea nitrogen	40 mg/dL	7 - 20 mg/dL
Serum creatinine	1.7 mg/dL	0.6 - 1.2 mg/dL
Estimated glomerular filtration rate	30 mL/min/1.73 m^2^	> 60 mL/min/1.73 m^2^

Chest X-ray and abdominopelvic computed tomography scan with intravenous contrast were unremarkable. COVID-19, influenzae A and B, a hepatitis panel, a bacterial gastrointestinal panel, and *Clostridium difficile *antigen/toxin testing were negative. A 2 L intravenous (IV) lactated Ringer’s bolus and subsequent infusion of normal saline were given. Blood and urine cultures were ordered, and empiric treatment with ceftriaxone and azithromycin was initiated for presumed community-acquired pneumonia.

Once admitted, labs revealed persistently elevated liver function enzymes, thrombocytopenia, and leukopenia. Additionally, lactic acid, procalcitonin, and creatinine kinase were elevated (Table 2). Infectious Disease was consulted to entertain the possibility of a tick-borne illness. The patient was transitioned to doxycycline 100 mg IV twice daily and cefepime to cover tick-borne illness and community-acquired pneumonia. Testing for tick-borne diseases was ordered with a peripheral blood smear review. Due to severe thrombocytopenia, with a platelet nadir of 15 K/uL, additional labs were ordered (Table 2), and she received one unit of platelets, doubling her count to 30 K/uL.

**Table 2:** Laboratory Values on Admission

Parameter	Result	Reference Range
D-dimer	> 20 ug/mL FEU	0.00 - 0.60 ug/mL FEU
Fibrinogen	274 mg/dL	243 - 550 mg/dL
Collagen/epinephrine closure	> 300 sec	86 -194 sec
Collagen/adenosine 5’ diphosphate time	> 219 sec	56 -110 sec
Lactate dehydrogenase (LDH)	753 U/L	100 - 190 U/L
Lactic acid	2.5 mmol/L	0.4 - 2.0 mmol/L
Procalcitonin	3.27 ng/mL	0.00 - 0.15 ng/mL
Total creatinine kinase	519 U/L	26 -192 U/L

On hospital day one, the peripheral blood smear was unremarkable for morulae. Overnight, the patient developed acute hypoxic respiratory failure, requiring oxygen via a non-rebreather mask. Repeat chest X-ray showed suspected mild interstitial edema, attributable to hypervolemia from fluids administered. She was shortly weaned off oxygen by morning. A bilateral lower extremity ultrasound venous duplex showed no evidence of deep vein thrombosis.

On hospital day two, her vitals and platelets remained stable, her AKI resolved, and her liver enzymes were improving. Her blood and urine cultures remained sterile. She was discharged with oral doxycycline.

After discharge, the polymerase chain reaction confirmed the presence of *A. phagocytophilum*. *Borrelia burgdorferi* testing showed prior infection with positive IgG and negative IgM.

At follow-up, testing showed a normal platelet count of 347 K/uL, stable renal function, and continued improvement in transaminases with ALT 98 U/L and AST 58 U/L.

## Discussion

Incidence of anaplasmosis has steadily increased in the United States according to the CDC [3]. This reported case is a part of that trend, but awareness needs to be spread to non-endemic areas. Ohio had zero reported cases of anaplasmosis from January through June 22nd of 2023 and 11 cases in all of 2022 [8]. Symptoms begin one to two weeks after infection, meaning physicians should be aware of imported cases from endemic areas and what early signs and laboratory studies could indicate tick-borne illnesses [9].

A retrospective chart review study from Hershey Medical Center found human granulocytic anaplasmosis infections in Pennsylvania rapidly rose from 2008 through 2021. They noted 61 cases at Hershey Medical Center alone over that time span, with the first case in 2013 and then a rapid rise from 2017 onward. By looking at the Pennsylvania Department of Health (DOH) data, they found cases were spreading geographically, with explosions in numbers in Eastern, Central, and Western counties of Pennsylvania. Less than 10 cases were reported in 2012 to the Pennsylvania DOH, while in 2021, there were more than 600 cases reported [9].

Similarities to this reported case were found in the Hershey Medical Center data of 61 patients. Of the reported cases, 75.4% were in those greater than 60 years of age. While only 39% recalled a tick bite, about 80% reported outdoor activity, which was the highest reported risk factor. Their most commonly presenting symptom was subjective fever (85%), which was a chief complaint for our patient. The study reported elevated liver enzymes and thrombocytopenia as common laboratory manifestations (71% and 69% of patients, respectively), and less often (27%) leukopenia, all three were seen in our patient [9]. Early empiric treatment is vitally important, as HGA can be deadly if untreated or if treatment is delayed. A systemic review of published cases identified 110 patients with HGA, in which six patients died. Of those six, two did not receive appropriate antibiotics, and another two did not receive them within the first 48 hours [10].

The Alleghany Public Health Department’s data revealed an uptick in county cases. From 2011 to 2020, they had a total of 27 cases, with zero cases in 2013 and 2015. Every year since 2018, the number of cases has been rising, with double-digit cases first starting in 2020 at a count of 13. Additionally, 2021 had 20 cases, 2022 had 33 cases, more than their 2011-2020 years combined. This seemingly compounding trend demonstrates the need for increased awareness of HGA amongst physicians as it is possibly now endemic or will be in the near future [11].

Other, more prevalent diseases, such as COVID-19 infection, can cause similar manifestations [12], making subjective and objective findings increasingly important. While anaplasmosis presents with a rash a minority of the time, it can present as a co-infection with other tick-borne diseases that frequently do, such as Lyme disease (LD) [10]. Using erythema migrans, the classic Bull’s-eye pattern rash from LD, to enroll patients from 1995 through 2004 in Valhalla, New York, one study found co-infection rates with HGA to be 2.3-10.0%, depending on the laboratory definition of a positive HGA infection [13]. Another study in Western Ukraine examined 498 patients from 2006 through 2014 who were treated for tick-borne illnesses, 60 of whom were diagnosed with HGA. Of those 60, 28 patients also had LD, equating to a HGA-LD co-infection rate of 46.7% (± 6.4%) [14].

## Conclusions

Due to the vague presentation, it is important to include human granulocytic anaplasmosis and other tick-borne diseases in a wide differential. Thrombocytopenia, leukopenia, and elevated liver enzymes should raise suspicion of a tick-borne etiology, even when not in endemic regions. This is becoming more important given the increase in areas and rates of the disease. Additionally, this could reduce diagnosis time and improve treatment outcomes when earlier intervention is initiated. 
